# The UL13 and US3 Protein Kinases of Herpes Simplex Virus 1 Cooperate to Promote the Assembly and Release of Mature, Infectious Virions

**DOI:** 10.1371/journal.pone.0131420

**Published:** 2015-06-26

**Authors:** Svetlana Gershburg, Joshua Geltz, Karin E. Peterson, William P. Halford, Edward Gershburg

**Affiliations:** 1 Department of Medical Microbiology, Immunology and Cell Biology, Southern Illinois University School of Medicine, Springfield, IL 62794–9626, United States of America; 2 Rocky Mountain Laboratories, National Institute of Allergy and Infectious Disease, Hamilton, MT 59840, United States of America; University of Regensburg, GERMANY

## Abstract

Herpes simplex virus type 1 (HSV-1) encodes two *bona fide* serine/threonine protein kinases, the *US3* and *UL13* gene products. HSV-1 ΔUS3 mutants replicate with wild-type efficiency in cultured cells, and HSV-1 ΔUL13 mutants exhibit <10-fold reduction in infectious viral titers. Given these modest phenotypes, it remains unclear how the US3 and UL13 protein kinases contribute to HSV-1 replication. In the current study, we designed a panel of HSV-1 mutants, in which portions of *UL13* and *US3* genes were replaced by expression cassettes encoding mCherry protein or green fluorescent protein (GFP), respectively, and analyzed DNA replication, protein expression, and spread of these mutants in several cell types. Loss of US3 function alone had largely negligible effect on viral DNA accumulation, gene expression, virion release, and spread. Loss of UL13 function alone also had no appreciable effects on viral DNA levels. However, loss of UL13 function did result in a measurable decrease in the steady-state levels of two viral glycoproteins (gC and gD), release of total and infectious virions, and viral spread. Disruption of both genes did not affect the accumulation of viral DNA, but resulted in further reduction in gC and gD steady-state levels, and attenuation of viral spread and infectious virion release. These data show that the UL13 kinase plays an important role in the late phase of HSV-1 infection, likely by affecting virion assembly and/or release. Moreover, the data suggest that the combined activities of the US3 and UL13 protein kinases are critical to the efficient assembly and release of infectious virions from HSV-1-infected cells.

## Introduction

Herpesviruses are an ancient group of double-stranded DNA viruses, which, due to their large genome size, encode a variety of accessory proteins including at least one serine/threonine protein kinase. While the biological functions of these viral protein kinases are not clear, these functions must be important at least due to the fact that despite having access to over 500 protein kinases encoded by the host cell, herpesviruses retained their protein kinases over the millennia as part of their core group of genes [1, 2]. The protein kinases encoded by herpesviruses fall into two groups: those conserved in all three herpesvirus subfamilies (α-, β-, and γ-) are termed “conserved herpesviral protein kinases” (CHPKs) [3], and the rest are present only in the neurotropic α-herpesviruses [4].

In human herpesviruses, the CHPKs include UL13 kinase of herpes simplex viruses types 1 and 2 (HSV-1 and -2), ORF47 kinase of Varicella Zoster Virus (VZV), UL97 kinase of human cytomegalovirus (HCMV), U69 kinase of human herpesviruses 6 and 7 (HHV-6 and -7), BGLF4 kinase of Epstein-Barr virus (EBV), and ORF36 kinase of Kaposi Sarcoma-associated herpesvirus (KSHV) [3, 5, 6]. Over the course of 25 years since their discovery, a number of studies have been performed to understand the role of CHPKs in replication of herpesviruses. When genes encoding for the CHPKs of human β- and γ- herpesviruses were knocked out [7–10] or their expression inhibited by RNAi [11, 12], replication of these viruses (or their fitness) was significantly impaired [7–12]. The replication defect appeared to occur at the nuclear egress level [7, 8, 11, 13] and the mechanism of this inhibition seemed to involve reduction in levels of nuclear egress complex (NEC) proteins ([7] and Gershburg, unpublished data). In contrast, studies involving CHPKs of α-herpesviruses (UL13 of HSV-1 and -2, and ORF47 of VZV) thus far yielded controversial data: several studies suggested that the UL13 kinase is dispensable for viral replication [14, 15], whereas others claimed that HSV-1 UL13-null viruses exhibit a 250-fold replication defect in certain cell lines [16]. Likewise, the conserved kinase of VZV, ORF47, has been found to either play an important role in viral replication in several cell types [17–19] or be dispensable for VZV replication [20]. Thus, the unifying theory that would explain what is the critical function of the CHPKs, which are highly conserved across a family of over 100 known herpesviruses is currently lacking.

The significance of the conserved UL13-like kinases in the life cycle of neurotropic α-herpesviruses is likely obscured by the fact that they all encode a second protein kinase, US3, acquired after separation of α-herpesviruses from β- and γ- herpesviruses [4]. The US3-like protein kinases might be involved in regulation of nuclear egress through the direct phosphorylation of nuclear lamina component lamin A/C [21], as well as the HSV-1 UL34 protein, a component of the nuclear egress complex [22–24], and glycoprotein B [25–27]. Yet this potential function does not translate into a defined role in viral replication and deletion of HSV-1's US3 gene has been reported to have either no effect on HSV-1 replication [28–32] or to inhibit replication by 10- to 30-fold [24, 33] in a cell type dependent manner.

In addition to these observable phenotypes, herpesvirus protein kinases phosphorylate a variety of substrates [3, 34] and are proposed to play a role in multiple processes crucial for viral replication. Specifically, the HSV-1 UL13 kinase phosphorylates infected cell protein (ICP) 22 [14, 15, 35], US3 protein kinase [36], tegument proteins UL41, UL49 [35], VP11/12 [37], glycoproteins I and E [38], and ICP0 [39, 40], as well as the cellular proteins p60 [41], the C-terminal domain of RNA polymerase II [16], suppressor of cytokine signaling-3 (SOCS3) [42], and translation elongation factor 1delta (EF-0031δ) [43]. Therefore, UL13 kinase has been proposed to play a role in regulation of ICP22 activity [44], stabilization and regulation of ICP0 activities [39, 40], regulation of VP11/12 activities [37], inhibition of the interferon signaling pathway [42, 45], repression of host transcription [46], and promotion of virion disassembly [47]. Likewise, the HSV-1 US3 kinase phosphorylates viral dUTPase [48], ICP22 [44], VP11/12 [37], glycoprotein B [25–27, 49], UL31 [50], UL34 [23], UL47 [51], ICP22 [44], p65/RelA [52], IRF3 [53], programmed cell death protein 4 (PDCD4) [54], tuberous sclerosis complex 2 (TSC2) [55], histone deacetylase 2 (HDAC2) [32], and Lamin A/C [21]. Consequently, the US3 kinase has been implicated in dampening NF-κB activation [52], resistance to interferon [53, 56, 57], regulation of viral replication and neuroinvasiveness through increase in dUTPase activity [58], stabilization of DNA sensor IFI16 [59], stabilization of microtubules [60], downregulation of MHC class I and immune evasion [61, 62], inhibition of TLR2 signaling [63], phospholipid synthesis [64], suppression of extracellular signal-regulated kinase (ERK) activity [65], inhibition of apoptosis [54, 66–77], stimulation of mRNA translation and viral replication [55], regulation of nuclear egress [21, 22, 24, 27, 33, 78–82], suppression of mitochondrial respiration [83], blocking of histone deacetylation [84], and attenuation of c-Jun N-terminal protein kinase (JNK) pathway [85].

This assortment of substrates and functions underscores a paradox in studies of α-herpesviral protein kinases: if these kinases perform so many important functions, why are the phenotypes of their mutants so modest? The obvious question one would ask: what is the phenotype of HSV-1 mutants lacking both US3 and UL13 kinases? Thus far, no published study focused on this question specifically and the only study describing such a mutant concluded that the phenotype of the double mutant is identical to phenotypes of single kinase mutants [15]. While such an outcome is possible, the stark difference between phenotypes of CHPK-knockout β- and γ- herpesviruses and the phenotype of double PK-knockout HSV-1 warrants a more focused study of the latter. Here, we compared side-by-side the fitness of a single kinase *vs* double kinase mutants generated on the background of a low-passage HSV-1 KOS [86]. We were interested in examining whether activities of UL13 and US3 kinases are redundant or complementary and whether the phenotype of the *ΔUS3/ΔUL13* mutant would be more appreciable then phenotypes of *ΔUS3* or *ΔUL13* mutants. Our data demonstrate that the HSV-1 *ΔUS3/ΔUL13* mutant exhibited defects in assembly and release of infectious virus which were more drastic than the combined defects of the HSV-1 *ΔUS3* or *ΔUL13* mutants. Our data suggest that combined activity of the HSV-1 protein kinases plays an important role in viral replication to the extent comparable to roles played by the CHPKs of β- and γ- herpesviruses in replication of these viruses.

## Materials and Methods

### Antibodies

Anti-ß-actin mouse mAb were purchased from Sigma-Aldrich (St. Louis, MO). The following antibodies were used for the detection of HSV-1 proteins: anti-ICP0 and anti-gD mouse mAb (Santa Cruz Biotechnology, Dallas, TX), anti-VP5 mouse mAb (Abcam, Cambridge, MA), rabbit anti-gC, anti-gG, anti-gB, and anti-gE were kindly provided by Drs. Cohen and Eisenberg (University of Pennsylvania), and anti-HSV-1 rabbit Ab (Dako Corporation, Carpinteria, CA). For immunoblotting, the secondary antibodies were: IRDye 800CW goat anti-mouse IgG and IRDye 680RD goat anti-rabbit IgG (LI-COR, Lincoln, NE).

### Cells and viruses

Vero, SK-N-SH, and HFF-1 cells were obtained from the American Type Culture Collection (ATCC). The cells were maintained in Dulbecco’s Modified Eagle’s medium (DMEM) supplemented with 5–10% fetal bovine serum (FBS) and antibiotic/antimycotic mixture (Invitrogen, Carlsbad, CA). Vero cell lines that stably express FLAG-tagged variants of HSV-1 US3 (US3^+^) and UL13 (UL13^+^) will be the topic of a forthcoming manuscript (A. Wilber, W. Halford, and E. Gershburg, unpublished data), and thus complete characterization of these cell lines is deferred. Briefly, a novel gene-delivery system was created based on a *Sleeping Beauty* (SB) transposable element that employs a bidirectional ICP0-L/ST promoter of HSV-1 [87]. One side of the promoter, which normally drives L/ST transcription [88], was engineered to drive expression of a genetic selection marker that encodes a truncated nerve growth factor receptor (tNGFR) [89]. The other side of the promoter, which normally drives ICP0 mRNA transcription, was engineered to drive expression of UL13-FLAG or US3-FLAG. Finally, the construct was modified to contain two Tet-operators immediately downstream of the TATA box where ICP0 mRNA transcription initiates to make mRNA synthesis TetR-repressible [90] and two SB direct repeats that flank the expression cassette [91]. Vero cells were co-transfected with three plasmids: (1) a SB-transposable cassette that co-expresses a selectable tNGFR marker and individual FLAG-tagged HSV-1 protein; (2) a SB-transposable cassette that co-expresses a Tet Repressor and a puromycin selection marker from an IRES-based bicistronic transcript driven by a CAGS promoter; and (3) a SB transposase expression vector. Following transient transfection, puromycin selection for stably transfected cells was initiated at 48 hours post-transfection and maintained for 3 weeks at which time fluorescence-activated cell sorting (FACS) was used to isolate a >99% pure population of tNGFR^+^ cells, which were cultured in the presence of puromycin.

The HSV-1 recombinant viruses used in this study were derivatives of early passage HSV-1 KOS [86]. All viral stocks were generated in Vero cells and, if necessary, were concentrated by ultracentrifugation.

### Plasmid precursors of HSV-1 recombinant viruses

The mutant ΔUS3 allele was generated as follows. Infectious HSV-1 KOS DNA was digested with *EcoR I* and *Avr II* restriction endonucleases and the fragment spanning bases 131,394–144,747 (Genbank #JQ673480) was subcloned into *EcoR I* and *Spe I* sites of pCR2.1 vector (Invitrogen, Carlsbad, CA). The resulting plasmid (termed p65) contained a significant portion of the unique short (Us) region of HSV-1 genome. Excess DNA sequences in p65 were removed by *Sac I* and *Stu I* digestion and ligated such that the resulting plasmid (termed p552) contained bases 131,395–137,540 of HSV-1 KOS. A fragment corresponding to amino acids 107 to 356 of US3 (bases 135,405–136,150) was excised by *BspE I* and *BamH I* digest and a cassette containing green fluorescent protein (GFP) coding sequence under the control of human phosphoglycerate kinase (PGK) promoter was ligated in its place. The resulting plasmid (p554) was digested with *Acc65 I* and a 3.5 kb fragment containing a mutant *ΔUS3* allele disrupted with a GFP expression cassette was subcloned into the *Kpn I* site of pCR2.1 to create p575.

The mutant *ΔUL13* allele was generated as follows. Fragments of the *UL13* gene (corresponding to bases 28,014–28,328 and 27,173–27,532) were PCR-amplified from HSV-1 KOS DNA and subcloned into pCR2.1 such that two fragments were bridged by a linker containing *Nde I*–*BamH I*–*Mlu I* restriction sites (p525). An expression cassette containing the mCherry coding sequence under the control of immediate-early CMV promoter was subcloned into *Nde I* and *Mlu I* sites of p525 and the resulting plasmid termed p529.

### Construction and isolation of HSV-1 recombinant viruses

To obtain HSV-1 infectious DNA, 3×10^7^ Vero cells were inoculated with 3 plaque forming units (pfu) per cell of HSV-1 strain KOS and incubated at 37°C. At 24 hours post-inoculation, cells were scraped, pelleted by centrifugation, rinsed with phosphate-buffered saline (PBS), and resuspended in 200mM EDTA (pH 8.0). Proteinase K (75μl of 10mg/ml) and SDS (375μl of 10% stock) were added to 7ml of the lysate and the mixture incubated for 16 hours at 50°C with slow rotation. Proteins were removed by repeated phenol/chloroform extractions; DNA was transferred into a dialysis bag (10,000MW cutoff; Pierce Chemical Co., Rockford, IL), dialyzed against 0.1× standard saline citrate (SSC) for 24 hours, aliquoted, and stored at -80°C until use.

Recombinant HSV-1 viruses were generated by homologous recombination. To this end, Vero cells (8×10^5^) were co-transfecting with 2μg of infectious HSV-1 KOS DNA and 1μg of each recombinant plasmid using TurboFect transfection reagent (Thermo Fisher Scientific, Pittsburgh, PA). After overnight incubation, the transfection medium was replaced with complete DMEM containing 1% methylcellulose and GFP^+^ or mCherry^+^ plaques were selected on the stage of a TE2000 fluorescent microscope (Nikon Instruments, Lewisville, TX). The GFP^+^ or mCherry^+^ recombinant viruses were passed in Vero cells until a uniform population of viruses was obtained.

### Southern blot analysis of US3 and UL13 loci in HSV-1 recombinant viruses

Vero cells were seeded at a density of 1.5×10^6^ cells per dish in 60-mm dishes and inoculated with 2.5 pfu per cell for 24 hours. Total DNA was isolated, digested with the restriction enzymes *Mlu I* and *Afl II*, and separated on 1% agarose gels. The DNA was transferred onto Zeta Probe GT nylon membranes (Biorad Laboratories, Hercules, CA), crosslinked by irradiation with 0.2J/cm^2^ in a UV crosslinker (Spectronics Corporation, Westbury, NY), and hybridized with radiolabeled oligonucleotide probes specific for the HSV-1 KOS *UL13* or the *US3* genes (S1 Table). Oligonucleotides were end-labeled with [α-^32^P] dATP using terminal deoxynucleotidyl transferase (Thermo Fisher Scientific, Pittsburgh, PA) and reacted with the nylon membranes for 16 hours at 42°C in a solution containing 5ng/ml labeled probe, 7% SDS, 120mM NaH_2_PO_4_, and 250mM NaCl. Excess probe was removed by sequential washes with 0.1× SSC containing 0.1% SDS. The hybridization patterns were visualized by exposure to phosphor screens, which were scanned and analyzed with a Cyclone PhosphorImager and OptiQuant software (Perkin Elmer, Boston, MA).

### Plaque assays

Vero cells were seeded at a density of 4×10^5^ cells per well in 6-well plates. After 6 hours, the cells were inoculated with log_10_-dilutions of HSV-1 KOS or each of the HSV-1 kinase mutants. Forty-five minutes after inoculation, the viral inoculum was replaced with complete DMEM containing 0.5% methylcellulose. Cell monolayers were fixed and stained with a solution of 20% methanol and 0.1% crystal violet at 72 hours post-inoculation, and plaques counted. For the microtiter plaque assay, Vero cells were seeded at a density of 2×10^4^ cells per well in 96-well plates and inoculated with 5-fold-dilutions of each of the viruses. The rest of the procedure was identical to the one described above.

For plaque size analyses, the plaques were processed 48 or 60 hours post-inoculation to minimize cell lysis. Cell monolayers were washed twice with 1× PBS to remove the methylcellulose and fixed in 3.7% formaldehyde. After permeabilization with 0.25% Triton X-100 (prepared in 1× PBS), cell monolayers were incubated for 30 min with PBS-F (0.5% bovine serum albumin in PBS) to block non-specific antibody binding. The blocking solution was replaced with rabbit anti-HSV-1 antibody (Dako Cytomation, Carpinteria, CA) diluted 1:1,000 for 2 h at room temperature. After additional washes the cell monolayers were incubated with Alexa Fluor 488-conjugated goat anti-rabbit secondary antibodies used at 1:1,000 dilution for 1 h. The plaques were imaged on TE2000 fluorescent microscope (Nikon Instruments, Lewisville, TX) and epifluorescence images were recorded with a Retiga Exi CCD (charge-coupled device) camera (QImaging).

### Immunoblotting

Vero cells (3×10^6^) were seeded in 60-mm dishes and inoculated with 2.5 pfu per cell for 14 hours. The cells were collected by treatment with 5mM EDTA (prepared in 1× PBS) and whole-cell lysates prepared by boiling for 5 minutes in Laemmli sample buffer [92]. Lysates equivalent to 1×10^5^ cells per lane were resolved by SDS/PAGE (10% gels), and transferred onto nitrocellulose membranes (Millipore). After incubation in blocking buffer [5% Difco skimmed milk (BD Biosciences) in TBST (Tris-buffered saline with 0.2% Tween-20)], the membranes were incubated overnight with mouse monoclonal antibodies against ICP0, glycoproteins gB, gC, gD, gE, and gG, and major capsid protein (VP5) diluted to 1:1,000 in blocking buffer. The membranes were washed in TBST and incubated for 1 hour with IRDye-800CW-conjugated goat anti-mouse secondary antibody (Li-Cor Biosciences) diluted 1:20,000 in blocking buffer. After additional washes with TBST, the proteins of interest were visualized on an Odyssey Infrared Imaging System (Li-Cor Biosciences). Equal loading of the samples was verified by re-probing the membranes with anti-β-actin mouse mAb diluted 1:2,000 in blocking buffer.

### Replication assays

Vero cells were seeded in 24-well plates at a density of 1.5×10^5^ cells per well. After 6 hours, the cells were inoculated with 0.1 (low MOI) or 2.5 (high MOI) pfu per cell. Forty-five minutes after inoculation, the viral inoculum was aspirated, cell monolayers were rinsed with 0.5ml of complete DMEM, and 1ml complete DMEM added to each culture. At indicated time points post-inoculation, the supernatants were transferred to new 24-well plates and the cells washed twice with 1× PBS and 1ml complete DMEM was added. Plates with both the supernatants and the cells were transferred to a −80°C freezer until use. Upon thawing, viral titers were determined by a microtiter plaque assay as described above.

### Dot-blot analyses of viral DNA synthesis

Vero cells were seeded in 24-well plates at a density of 1.5×10^5^ cells per well. After 6 hours, the cells were inoculated with 2.5 pfu per cell with HSV-1 KOS, or HSV-1 Δ*UL13*/Δ*US3*, Δ*US3*, and Δ*UL13* mutants. As a positive control for inhibition of viral DNA sequences, a subset of HSV-1 KOS-infected cultures was treated with 300μM acyclovir (ACV), a known inhibitor of viral DNA synthesis. At 9, 12, 15, 18, 21, and 24 hours after inoculation, cells were lysed in 0.5ml of 0.4M NaOH/10mM EDTA, denatured at 95°C, snap-cooled on ice, and directly blotted on Zeta Probe GT nylon membrane (Bio-Rad Laboratories, Hercules, CA) in an 8×12 dot-blot pattern using a Convertible vacuum filtration manifold (Whatman-Biometra, Gröningen, Germany). DNA was crosslinked by irradiation with 0.2J/cm^2^ in a UV crosslinker (Spectronics Corporation, Westbury, NY) and membranes hybridized to a ^32^P-labeled oligonucleotide probe specific for the HSV-1 *US6* gene (S1 Table). The membranes were washed in 0.1× SSC/0.1% SDS, exposed to phosphor screens, which were scanned and analyzed with a Cyclone PhosphorImager and OptiQuant software (Perkin Elmer, Boston, MA).

### Sucrose gradient purification of the HSV-1 virions

Vero cells were seeded at a density of 7×10^6^ cells per 100 mm dish and inoculated with 2.5 pfu per cell of HSV-1 KOS, or HSV-1 Δ*UL13*/Δ*US3*, Δ*US3*, and Δ*UL13* mutants. Cells infected with HSV-1 KOS and treated with 300μM acyclovir (ACV) served as a replication-deficient control, whereas uninfected Vero cells served as background. At 18 hours post-inoculation, culture media was removed and stored at -80°C until use. Following removal of cell debris by low speed centrifugation (2,000×g for 5 minutes), samples were loaded on a discontinuous gradient consisting of 5 ml 15% sucrose, 5 ml 35% sucrose, and 5 ml 60% sucrose. Samples were centrifuged in a Beckman SW27 rotor at 25,000 rpm for 3 hours, and twenty ~0.5 ml sucrose fractions collected by piercing the bottom of each centrifuge tube with a 25G needle. Sucrose concentration in each fraction was measured by determining the refractive index using a refractometer (Atago, Japan), and relative abundance of HSV-1 virions was analyzed by ELISA as follows. Each fraction was diluted 1:5 in sodium carbonate buffer (pH– 9.6) and used as coating antigen in duplicate wells of a high binding 96-well enzyme immunoassay plate (Corning, Corning, NY). After overnight incubation at 4°C, HSV-1 virion-containing fractions were discarded, the wells were blocked with 2% dry milk in PBS/0.05% Tween-20 (Sigma-Aldrich, St Louis, MO) for 1 hour and a 1:5,000 dilution of a horseradish peroxidase-conjugated rabbit anti-HSV-1 antibody (Dako Cytomation, Carpinteria, CA) was added to each well. After a 1-hour incubation, excess antibody was removed by extensive washes with PBS/0.05% Tween-20, and 1-Step Ultra TMB substrate (Thermo Fisher Scientific, Pittsburgh, PA) was added to each well. Color development was measured at OD_655_ nm using a plate reader (Bio-tek Instruments, Winooski, VT). Relative virion yield in each group was characterized using two values: (1) area under the curve (AUC) used to describe the total amount of viral antigen in all fractions, and (2) peak value (PV) used to describe the highest level of viral antigen in a single fraction. Relative yields were expressed as OD_655_ absorbance levels with subtraction of background defined by levels obtained from uninfected Vero cell culture medium fractions. The infectivity in sucrose fractions was measured as follows: 100 μl of each fraction corresponding to 25%-35% and 35%-45% sucrose concentration ranges were pooled and the infectivity of the virions in these pooled fractions was measured by standard plaque assays in 96-well plates in Vero cells as described earlier in this section.

### Electron microscopy

Vero cells were seeded on Thermonox coverslips (Thermo Scientific) at a density of 2×10^5^ cells per well in a 24-well plate and inoculated with 2.5 pfu per cell of HSV-1 KOS, or HSV-1 Δ*UL13*/Δ*US3*, Δ*US3*, and Δ*UL13* mutants for 16 hours. The cells were washed with 1× PBS, fixed in 2.5% glutaraldehyde (pH 7.4) (Electron Microscopy Sciences, Hatfield, PA) and then post fixed in 1%OsO_4_/1.5% K_4_Fe(CN)_6_ in 0.1M sodium cacodylate in microwave under vacuum (2 min on, 2 min off, 2 min on). The samples were then stained with 1% tannic acid and en-bloc stained with 2% uranyl acetate under the same conditions. The samples were dehydrated in EtOH and embedded in Epon-Araldite mixture (Embed 812) (Electron Microscopy Sciences, Hatfield, PA). The samples were imaged on a Hitachi H-7500 transmission electron microscope (Hitachi, Dallas, TX) at 80 kV. Images were acquired with a Hamamatsu XR-100 digital camera system (AMT, Woburn, MA). Separate images of the nucleus, cytoplasm and plasma membranes/extra cellular space were recorded for 20 individual cells per each virus. The number of nucleocapsids and virions were manually counted in each image in a blinded study. The outliers were detected by performing Grubb’s test using an online GraphPad outlier calculator (http://graphpad.com/quickcalcs/Grubbs1.cfm).

### Statistical analysis of data

Statistical analyses were performed with Microsoft Excel (Microsoft Corporation, Redmond, WA) and GraphPad Prism (GraphPad Software, Inc., La Jolla, CA). Unless otherwise indicated, all data are presented as the mean ± standard error of the mean (sem). The significance of differences between multiple groups was compared by one-way analysis of variance (ANOVA) followed by Tukey’s post hoc t-test.

## Results

### Construction of HSV-1 UL13 and US3 kinase mutants

**Fig 1:**
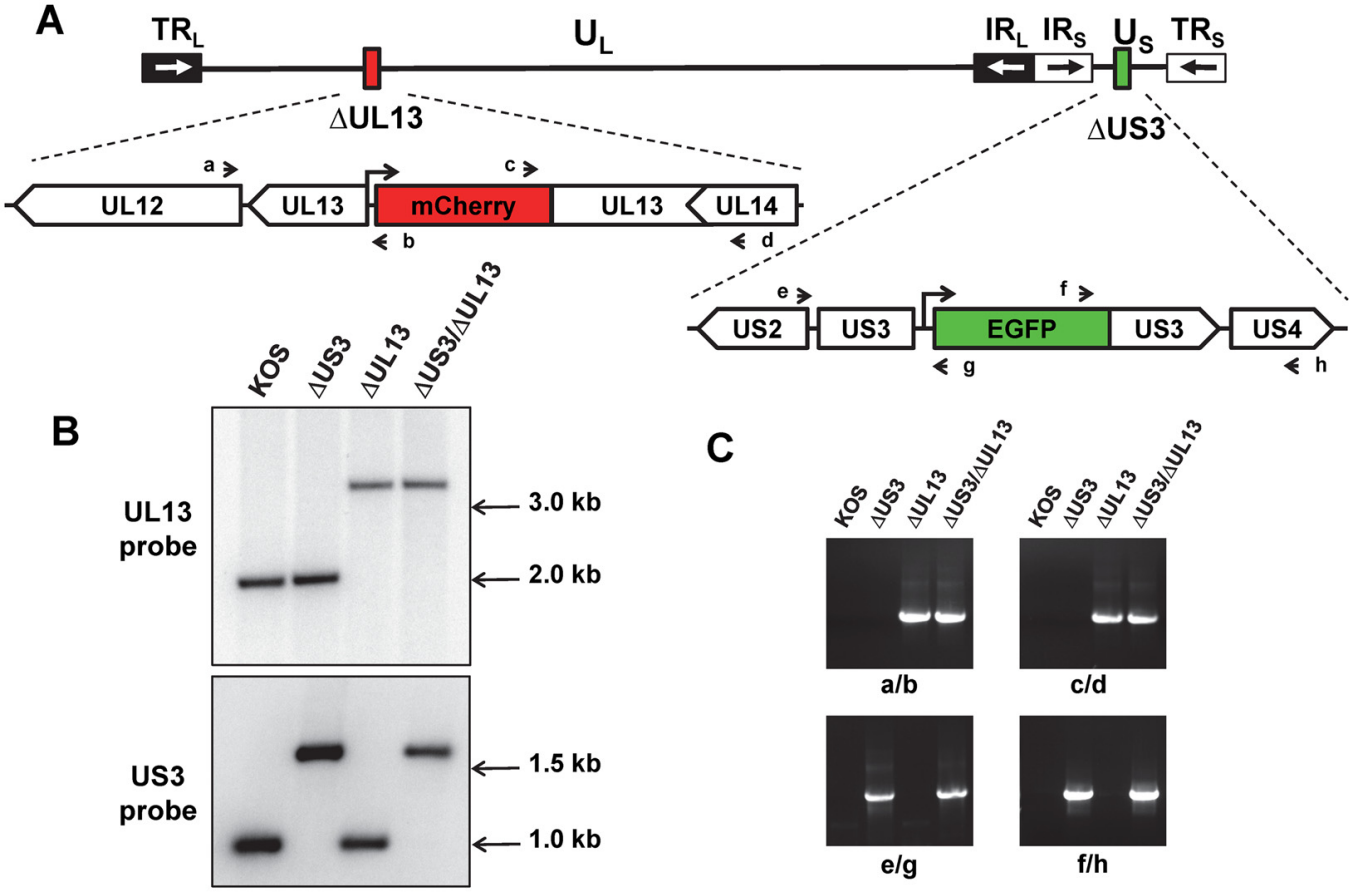
Construction and analyses of mutant US3 and UL13 alleles. (A) Schematic representation of the HSV-1 genome. Unique DNA sequences are represented by horizontal lines, and inverted repeats flanking the unique sequences are shown as open and black boxes with black and white arrows, respectively. US3 and UL13 regions where mutant alleles were generated are enlarged and position of the mCherry and GFP cassettes shown as red and green boxes, respectively. The approximate positions of primers designated a, b, c, d, and e, f, g, h used for PCR analyses are shown as short black arrows. (B) Southern blotting analyses of HSV-1 *ΔUS3*, *ΔUL13*, and *ΔUL13/ΔU3* mutants. Viral DNA preparations from Vero cells infected with wild type KOS or mutant viruses were digested with *Afl*II (top panel) and *Mlu*I (bottom panel) and subjected to Southern blotting. Membranes were probed with ^32^P-labeled UL13- and US3-specific oligonucleotide probes (S1 Table). Positions of DNA size standards are shown on the right. (C) PCR analyses of HSV-1 *ΔUS3*, *ΔUL13*, and *ΔUL13/ΔUS3* mutants. Total DNA isolated from Vero cells infected with wild type HSV-1 KOS or mutant viruses were used as template for PCR reactions with 4 sets of primers. In each set, one primer is located outside of the region involved in homologous recombination and another primer is located within the mCherry or GFP cassettes. The PCR reactions were resolved on 1.2% agarose gels and visualized by ethidium bromide staining.

HSV-1 mutant viruses lacking portions of the *UL13* or *US3* genes were constructed by homologous recombination between infectious wild-type HSV-1 KOS DNA and plasmids carrying mCherry or GFP selection markers, respectively (Fig 1A). The infectious wild-type HSV-1 KOS DNA was prepared from a virus stock 12 passages removed from the original clinical isolate [86]. A plasmid bearing *UL13* arms separated by a CMV promoter-*mCherry* reporter gene was recombined into HSV-1 KOS to yield a Δ*UL13* virus in which codons 151–303 of the *UL13* gene were deleted (Fig 1A). Likewise, a plasmid bearing US3 arms separated by a PGK promoter-*GFP* reporter gene was recombined into HSV-1 KOS to yield a Δ*US3* virus in which codons 107–356 of the *US3* gene were deleted (Fig 1A). The HSV-1 Δ*UL13/ΔUS3* mutant virus was constructed by homologous recombination between infectious HSV-1 Δ*UL13* DNA and the plasmid containing the ΔUS3 allele and GFP selection marker (Fig 1A). The insertion of mCherry and GFP expression cassettes into the anticipated loci in the HSV-1 genome was independently verified by Southern blot analysis and PCR (Fig 1B and 1C). Southern blot analysis confirmed that each HSV-1 mutant virus carried an insertion of the expected size and restriction fragment length polymorphism within the *UL13* and *US3* loci (Fig 1B). Finally, PCR analysis confirmed the presence of the *mCherry* reporter gene in the *UL13* locus of both the *ΔUL13* and *ΔUL13/ΔUS3* mutants, and reciprocally confirmed the presence of the *GFP* reporter gene in the *US3* locus of the *ΔUS3* and Δ*UL13*/Δ*US3* mutants (Fig 1C). Collectively, these data indicated that the intended mutations were successfully introduced into the HSV-1 genome.

### A HSV-1 *ΔUL13/ΔUS3* mutant forms micro-plaques that are smaller than plaques formed by HSV-1 *ΔUL13* or *ΔUS3* mutants

Three independent recombinants were generated for each HSV-1 mutant. Independent isolates of the HSV-1 Δ*US3* mutant produced plaques that were equivalent in size to wild-type HSV-1 KOS. In contrast, the HSV-1 Δ*UL13* isolates produced smaller plaques relative to wild-type HSV-1 KOS, which is consistent with previous reports [15, 93]. Moreover, all independent isolates of the HSV-1 Δ*UL13*/Δ*US3* mutant virus produced micro-plaques which appeared smaller than the plaques produced HSV-1 Δ*UL13* mutant.

**Fig 2:**
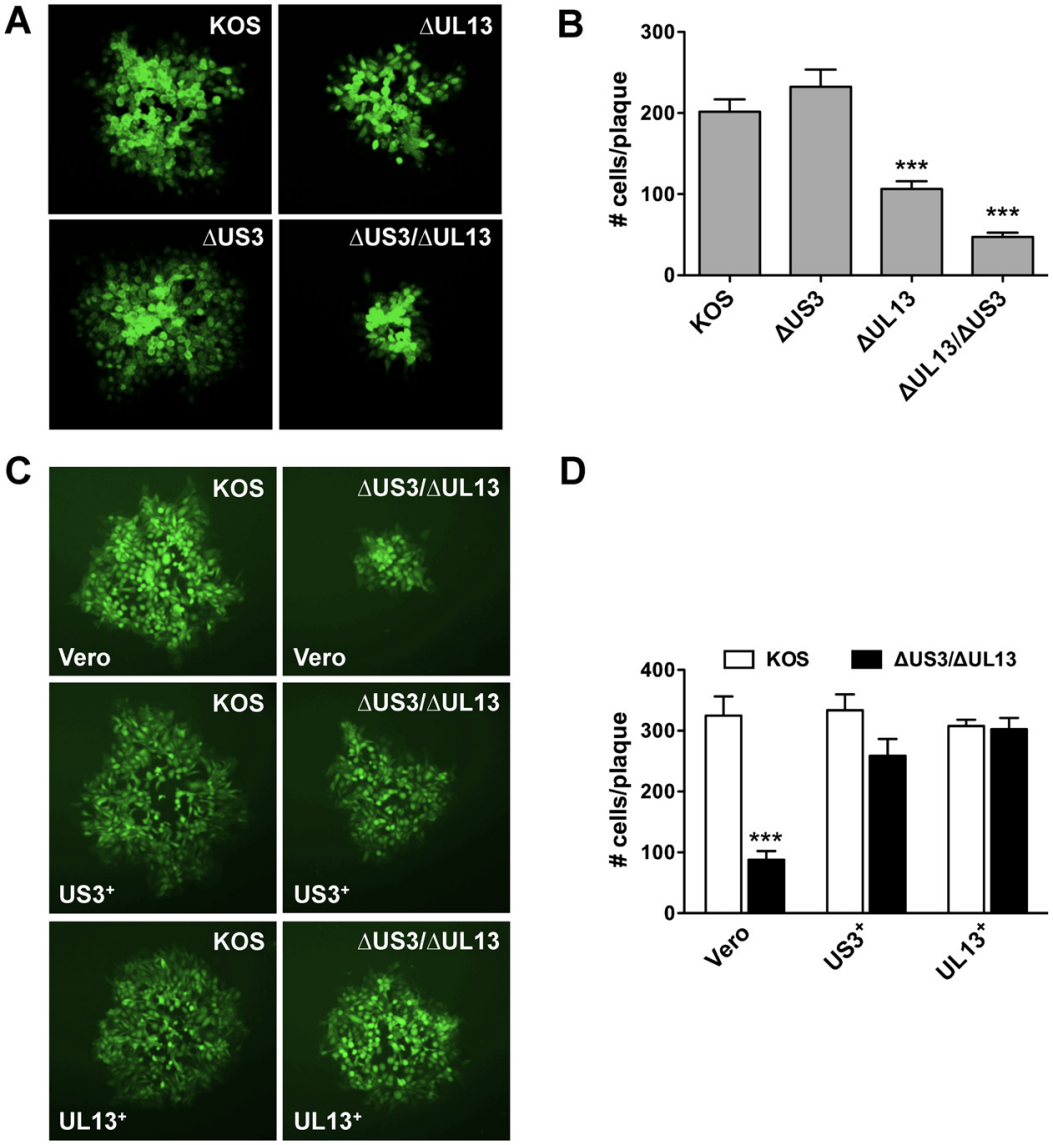
HSV-1 ΔUL13 mutants exhibit ‘small-plaque’ phenotype. (A) Vero cells and (C) US3^+^- and UL13^+^- cells were inoculated with 300 pfu of HSV-1 KOS or HSV-1 kinase mutants. At 48 (A) and 60 (C) hours post-inoculation, the monolayers were fixed and labeled with FITC-conjugated anti-HSV-1 antibody. Representative plaques are shown at magnification of 100×. (B) and (D) Ten plaques in each infection were photographed, and infected cells were enumerated. Results are presented as the mean number of infected cells per plaque ± sem (n = 10 plaques; ***—*p*<0.001).

To formally determine if the HSV-1 Δ*UL13*/Δ*US3* mutant exhibited greater defect in viral spread relative to the HSV-1 Δ*UL13* or Δ*US3* mutants, monolayers of Vero cells were inoculated with 300 pfu of either HSV-1 KOS, or HSV-1 Δ*UL13*, Δ*US3*, and Δ*UL13*/Δ*US3* mutants and overlaid with medium containing 0.5% methylcellulose. At 48 hours post-inoculation, the monolayers were fixed and immunofluorescently labeled with FITC-conjugated polyclonal HSV-1 antibody. Ten plaques were randomly selected per culture, photographed (Fig 2A), and the number of infected cells per plaque enumerated (Fig 2B). Plaques formed by wild-type HSV-1 KOS contained 202 ± 15 viral antigen-positive cells, and loss of US3 function alone (Δ*US3*) did not significantly alter plaque size (232 ± 21) (Fig 2B). Loss of UL13 function alone (Δ*UL13*) reduced the number of infected cells per plaque by ~50% (106 ± 10) relative to HSV-1 KOS (Fig 2B; *p*<0.0001). Consistent with our observations during plaque purification, loss of both UL13 and US3 kinases further reduced HSV-1 plaque size to 20% of wild-type HSV-1. Specifically, plaques formed by the HSV-1 Δ*UL13*/Δ*US3* mutant contained 47 ± 5 viral antigen-positive cells, and this reduction was significant relative to HSV-1 KOS (*p*<0.0001), the HSV-1 *ΔUS3* mutant (p<0.0001), and the HSV-1 Δ*UL13* mutant (Fig 2B; *p*<0.05).

To verify whether the observed defects in spread were due to the lack of the viral protein kinases, monolayers of Vero, UL13^+^-, and US3^+^- cells were inoculated with HSV-1 KOS or HSV-1 Δ*UL13*/Δ*US3* mutant as described above and the number of infected cells per plaque enumerated at 60 hours post-inoculation (Fig 2D). Consistent with the previous assay, plaques formed by the HSV-1 Δ*UL13*/Δ*US3* mutant were ~75% smaller than the wild-type HSV-1 KOS plaques on Vero cells (88 ± 15 *vs* 325 ± 32, respectively) (Fig 2D, *p*<0.0001). The US3 kinase provided *in trans* increased the number of infected cells in plaques formed by the HSV-1 Δ*UL13*/Δ*US3* mutant to 259 ± 28 relative to 334 ± 26 for the wild-type HSV-1 KOS (Fig 2D). Finally, in UL13^+^- complementing cells, the wild-type HSV-1 KOS and the HSV-1 Δ*UL13*/Δ*US3* mutant formed plaques of essentially identical size (307 ± 10 *vs* 302 ± 19, respectively) (Fig 2D). These data confirmed that defective phenotype observed during replication of the HSV-1 Δ*UL13*/Δ*US3* mutant is due to the lack of UL13 and US3 protein kinases and can be complemented by providing these kinases *in trans*.

Collectively, these results supported the hypothesis that the UL13 and US3 protein kinases might act in a cooperative manner to support HSV-1 spread in Vero cells; hence, loss of function of the *UL13* kinase *or* the *US3* kinase only partially reveals the magnitude of spread deficiency observed with a HSV-1 Δ*UL13*/Δ*US3* mutant.

### HSV-1 DNA accumulation is not affected by the absence of the UL13 or US3 kinases

A number of published hypotheses suggest that the UL13 and US3 kinases play important roles in both the immediate-early/early phase and the late phase of the HSV-1 life cycle [16, 32, 39, 40, 44, 59, 84]. Thus, we chose first to identify phases of HSV-1 replication at which the loss of UL13, US3, or both protein kinases would generate a measurable effect. Our initial experiment focused on testing a null hypothesis that "loss of the UL13 and US3 protein kinases would have no effect on the kinetics of HSV-1 DNA accumulation in virus-infected cells". If this null hypothesis was correct, it would suggest that the Δ*UL13*/Δ*US3* mutant's micro-plaque phenotype was most likely due to defects in the late stages of HSV-1 replication (i.e., after viral DNA synthesis). In contrast, significant changes in HSV-1 DNA accumulation would suggest that the UL13 and US3 protein kinases play a role in the immediate-early and early phases of HSV-1 replication (prior to viral DNA synthesis).

**Fig 3:**
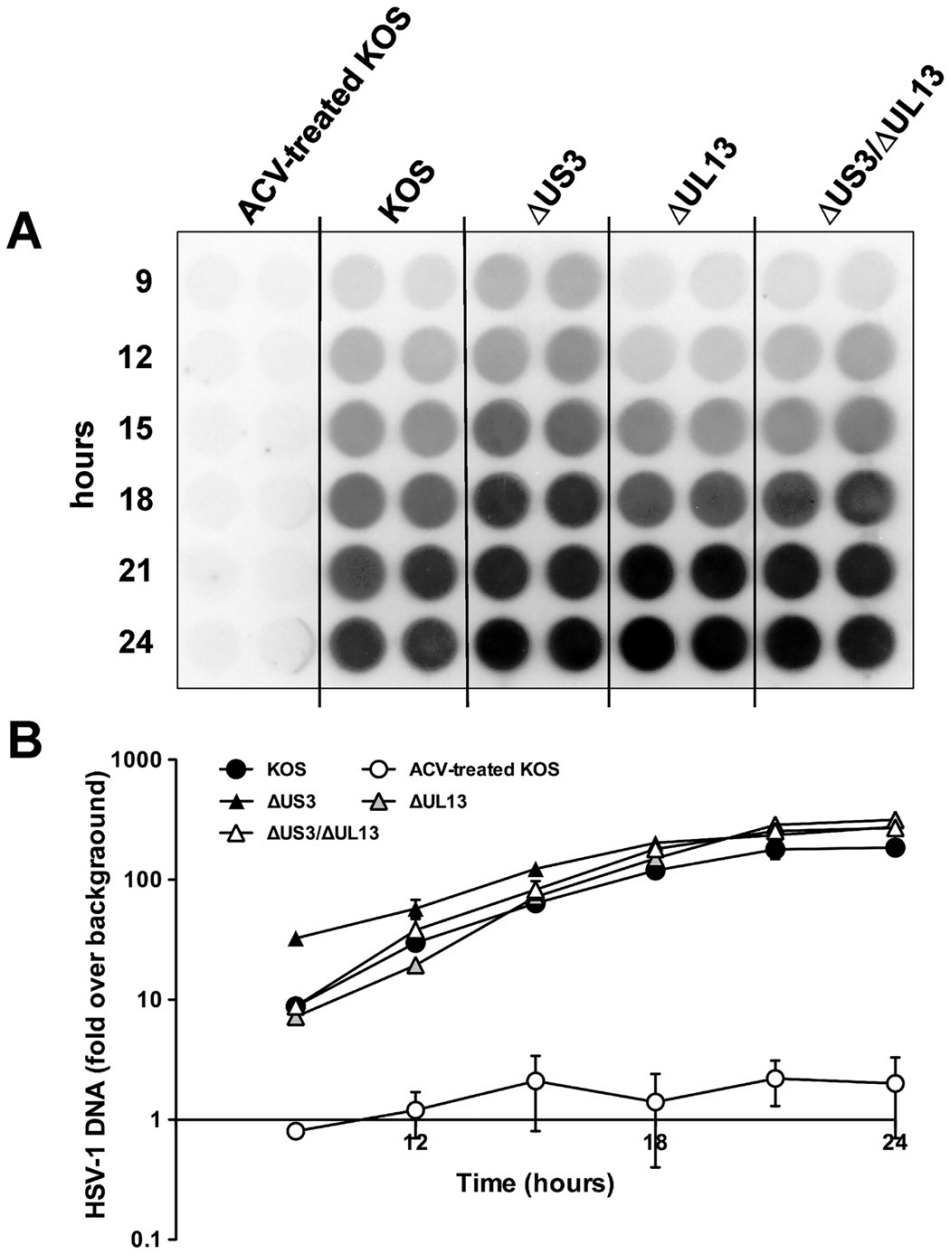
Viral DNA accumulation during the infection with HSV-1 KOS or *HSV-1* kinase mutants. (A) Vero cells were inoculated with wild type HSV-1 KOS or HSV-1 *ΔUS3*, *ΔUL13*, and *ΔUL13/ΔUS3* mutants at an MOI of 2.5 pfu per cell. At indicated time points, lysates were prepared and blotted onto a nylon membrane. The accumulation of viral DNA was assayed by hybridization to a ^32^P-labeled *US6*-specific oligonucleotide probe. ACV-treated KOS served as a negative control. (B) Relative intensity of hybridization signals was quantified by phosphorimager analysis. Results are shown as fold change in hybridization signal intensity relative to the background ± standard deviation (SD) of two duplicate infections.

To differentiate between these two possibilities, HSV-1 DNA accumulation was compared in Vero cells inoculated with 2.5 pfu per cell of either HSV-1 KOS, or HSV-1 Δ*UL13*, Δ*US3*, and Δ*UL13*/Δ*US3* mutants. Uninfected (UI) Vero cells were included as a negative control, and an additional group of Vero cells were inoculated with HSV-1 KOS in the presence of 300 μM acyclovir (ACV) served as a positive-control for inhibition of viral DNA synthesis. DNA samples were isolated at 3-hour intervals between 9 and 24 hours post inoculation, immobilized on a nylon membrane, and hybridized to an HSV-1-specific probe (Fig 3A). PhosphorImager analysis of the dot-blot indicated that viral DNA yields and the kinetics of viral DNA accumulation were similar in cells inoculated with HSV-1 KOS, or HSV-1 Δ*UL13* and *ΔUL13/ΔUS3* mutants (Fig 3A). In cells inoculated with HSV-1 *ΔUS3* mutant, viral DNA accumulated to ~ 6-fold higher levels at 9 hours post-inoculation, but was similar to other tested viruses at later time points. Viral DNA accumulation in cells inoculated with all tested viruses peaked between 18 and 24 hours post-inoculation and reached levels that were 170 to 260-fold higher than uninfected Vero cells (Fig 3B). In contrast, HSV-1 KOS-infected cells treated with ACV exhibited levels of viral DNA accumulation that did not significantly differ from the uninfected Vero cells (Fig 3B).

These data were consistent with a null hypothesis that loss of US3 kinase, UL13 kinase, or both did not significantly alter the kinetics of viral DNA accumulation relative to Vero cells infected with the wild-type HSV-1. Therefore, the UL13 and/or US3 kinases appear to be dispensable for the immediate-early and early phases of HSV-1 replication, implying that the defect in virus spread in cells infected with an HSV-1 Δ*UL13*/Δ*US3* mutant (Fig 2) occurs in the late phase of HSV-1 replication.

### Absence of UL13 kinase alters the accumulation of HSV-1 glycoproteins C and D

**Fig 4:**
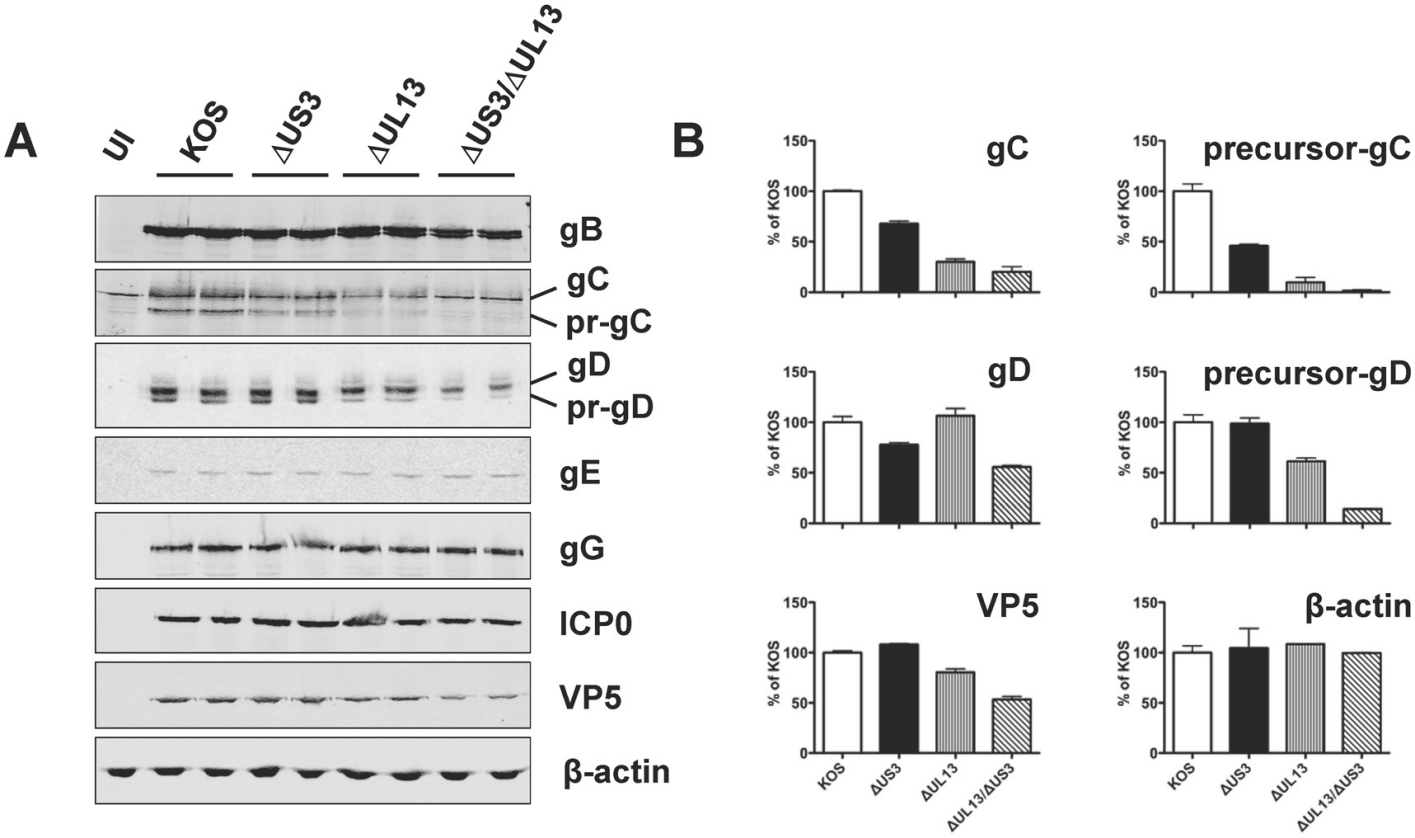
Expression of viral proteins in Vero cells infected with HSV-1 KOS or *ΔUS3* and *ΔUL13* mutants. (A) Vero cells were inoculated in duplicates as described in Fig 3. At 14 hours post-inoculation, whole-cell lysates were prepared and resolved by SDS-PAGE followed by immunoblot analyses with respective antibodies. Each lane contains an amount of lysate equal to 10^5^ cells. Levels of β-actin served as a loading control. (B) The relative intensity of signals was quantified by densitometry using ImageJ [106]. Results are shown for proteins whose levels of expression changed substantially as percent change in signal intensity relative to the wild type (HSV-1 KOS) (set to 100) ± SD of the duplicate infections.

To verify the effects of loss of the US3 or UL13 kinases on early and late stages of HSV-1 replication, expression levels of several viral genes were compared. Vero cells were inoculated with 2.5 pfu per cell of HSV-1 KOS, or HSV-1 kinase mutants. At 14 hours post-inoculation, whole-cell lysates were prepared and subjected to SDS-PAGE and immunoblotting analyses, and expression levels of several HSV-1 proteins were quantified by densitometry. Uninfected (UI) Vero cells were included as a negative control (Fig 4). The steady-state levels of the ICP0 protein (expressed from an immediate-early gene) were nearly identical across all viruses (Fig 4A). Likewise, the steady-state levels of another immediate-early protein, ICP4, were equal in cells inoculated with all tested viruses (not shown). Among the proteins expressed from HSV-1 late genes, the steady-state levels of glycoproteins B, E, and G were not significantly affected by the loss of US3 and UL13 protein kinases. In contrast, the steady-state levels of glycoprotein C (gC) were reduced more significantly: the levels of precursor gC (pr-gC) decreased 2-fold during the infection with the Δ*US3* mutant, 10-fold during the infection with the Δ*UL13* mutant, and were essentially undetectable during the infection with the Δ*UL13*/Δ*US3* mutant (Fig 4B). Consequently, the steady-state levels of mature gC were reduced by 3- and 5-fold for the Δ*UL13* and Δ*UL13*/Δ*US3* mutants, respectively. Likewise, during the infection with the Δ*UL13*/Δ*US3* mutant, the steady-state levels of glycoprotein D (gD) were reduced such that the levels of precursor gD (pr-gD) were 7-fold lower and mature gD 2-fold lower relative to the HSV-1 KOS (Fig 4B). These results correlate with the observation of a small-plaque phenotype exhibited by the UL13-deficient HSV-1 mutants (Fig 2) and corroborate the viral DNA accumulation data (Fig 3), suggesting that the HSV-1 *ΔUL13* mutants are deficient in their ability to spread from one host cell to another and that the activity of viral protein kinases is crucial at the late phase of viral infection. Notably, two of the tested viral proteins, ICP0 and gE, are reported substrates of the UL13 protein kinase [38–40], yet the loss of UL13 kinase had no effect on the steady-state levels of these proteins (Fig 4A).

### HSV-1 ΔUL13/ΔUS3-infected cells are defective for infectious virion release

**Fig 5:**
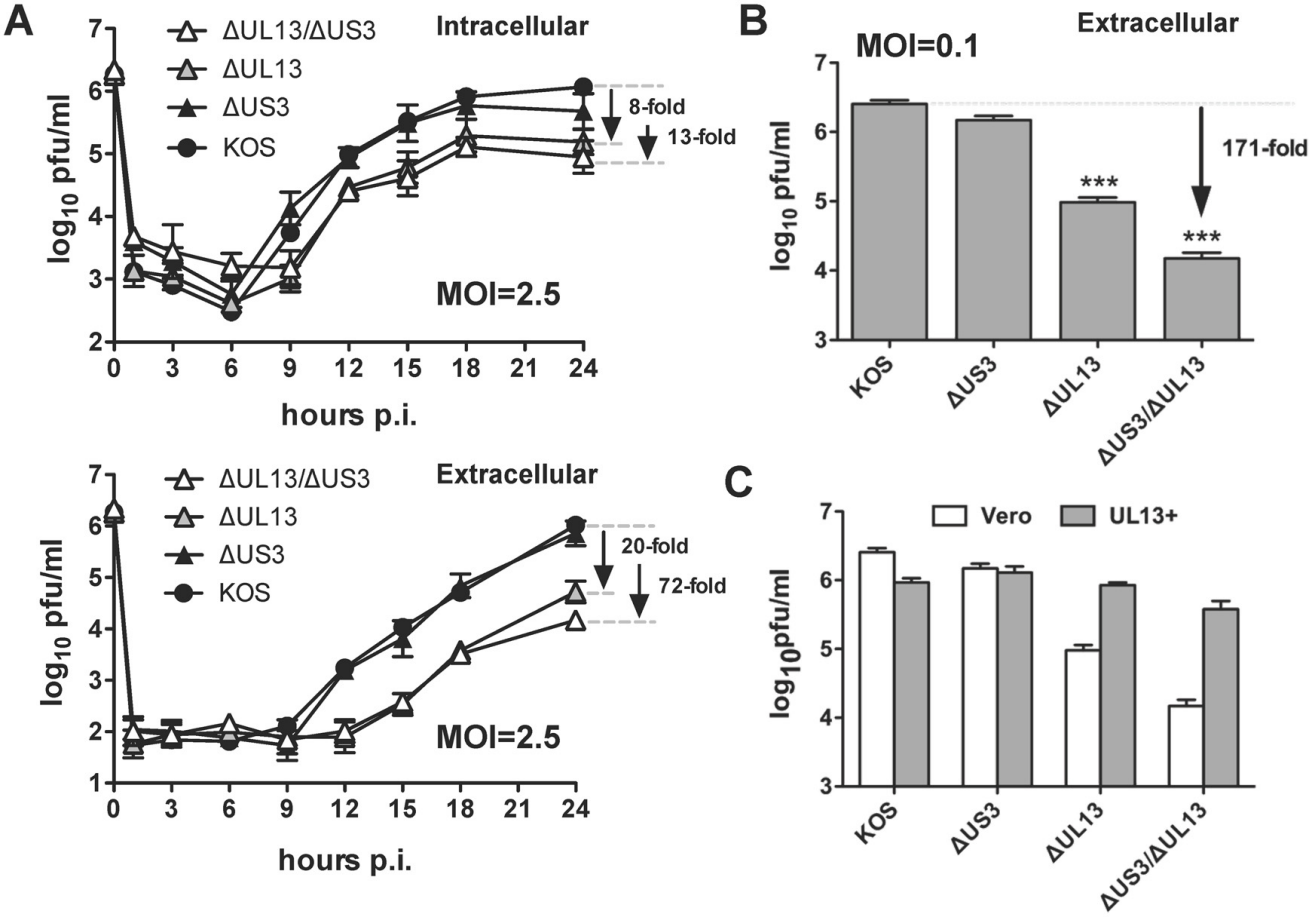
The yields of extracellular virus are significantly reduced in Vero cells infected with HSV-1 *ΔUL13* mutants. (A) Vero cells were inoculated with wild type HSV-1 KOS or HSV-1 kinase mutants at MOIs of 2.5 pfu per cell. Viral yields were determined by plaque assay in culture supernatants (extracellular) and cell lysates (intracellular) at 1, 3, 6, 9, 12, 15, 18, and 24 hours post-inoculation. (B) Vero cells were inoculated with wild type HSV-1 KOS or HSV-1 kinase mutants at MOIs of 2.5 pfu per cell. Viral yields were determined by plaque assay in culture supernatants (extracellular) collected at 36 hours post-inoculation. (C) Vero and UL13^+^- cells were inoculated with the HSV-1 *ΔUL13/ΔUS3* mutant at an MOI of 0.1 pfu per cell. Viral yields were determined by plaque assay in culture supernatants (extracellular). For all panels, results are presented as the mean virus yield ± sem (n = 6; ***—*p*<0.001).

To examine whether the activity of UL13 and US3 kinases contribute to virion morphogenesis or release of infectious virions, titers of intracellular and extracellular infectious virions were compared in Vero cells. The cells were inoculated with 2.5 pfu per cell of HSV-1 KOS, or HSV-1 Δ*UL13*/Δ*US3*, Δ*US3*, and Δ*UL13* mutants. The titers of intracellular infectious virus in cell monolayers and extracellular infectious virus in culture supernatants were measured at 1, 3, 6, 9, 12, 15, 18, and 24 hours post-inoculation by plaque assay (Fig 5A). Loss of US3 kinase alone (Δ*US3*) had only a negligible effect (2-fold decrease) on titers of the intracellular and extracellular infectious virions relative to the wild-type HSV-1 KOS (Fig 5A). Loss of UL13 kinase alone (Δ*UL13*) yielded an 8-fold decrease in titers of the intracellular virus relative to KOS by 24 hours post-inoculation, but decreased the titers of the extracellular virus by 20-fold relative to KOS (Fig 5A). Moreover, loss of both US3 and UL13 kinases (Δ*UL13*/Δ*US3*) yielded a 13-fold decrease in titers of the intracellular virus relative to KOS and a 72-fold decrease in titers of the extracellular virus relative to KOS (Fig 5A). The titers of the *ΔUL13* mutant viruses started to lag between 9 hours post-inoculation for the intracellular virions to 12 hours post-inoculation for the extracellular virions (Fig 5A). The titers of the extracellular infectious virions decrease even more severely when Vero cells were inoculated with a low MOI of 0.1 pfu per cell. Measured at 36 hours post-inoculation, the titers of the ΔUL13 mutants decreased 27-fold (for the HSV-1 *ΔUL13* mutant) and 171-fold (for the HSV-1 *ΔUL13/ΔUS3* mutant) relative to the wild-type HSV-1 KOS (Fig 5B).

To verify whether the defective phenotype of these mutants is due to the loss of UL13 kinase, the yields of the extracellular infectious virions were compared in Vero and UL13^+^-complementing cells (Fig 5C). The HSV-1 KOS and *ΔUS3* mutant replicated in both cell lines with comparable efficiency (KOS titers were reduced about 3-fold in UL13^+^ cells). In contrast, in Vero cells replication of both the *ΔUL13* and the *ΔUL13/ΔUS3* mutants was attenuated about 30- and 175-fold, respectively, relative to the wild-type virus. However, in the UL13^+^-complementing cells, the replication of these mutants was only ~ 2-fold lower than that of the wild-type virus (Fig 5C). These results show that expression of the UL13 kinase *in trans* fully complemented replication of the *ΔUL13* and Δ*UL13*/Δ*US3* mutants restoring levels of the extracellular virus to the wild type HSV-1 KOS levels (Fig 5C) and suggesting that the observed phenotype is due to the lack of viral protein kinases.

Collectively, these data suggest that UL13 kinase function is more important for virion release since its loss results in a more pronounced reduction in levels of extracellular virus (almost 30-fold at low MOI). Importantly, functions of both kinases appear to be critical for optimal production of infectious virions since the loss of both US3 and UL13 kinases resulted in marked reduction of infectious virion titers (Fig 5), which was larger than combined reductions in titer for each of the mutants lacking a single kinase. Finally, these data are highly consistent with the small-plaque phenotypes reported for the HSV-1 Δ*UL13* and Δ*UL13*/Δ*US3* mutants in Fig 2.

### Loss of HSV-1's UL13 kinase, but not US3 kinase, significantly decreases virion release from virus-infected cells: sucrose gradient analysis

**Fig 6:**
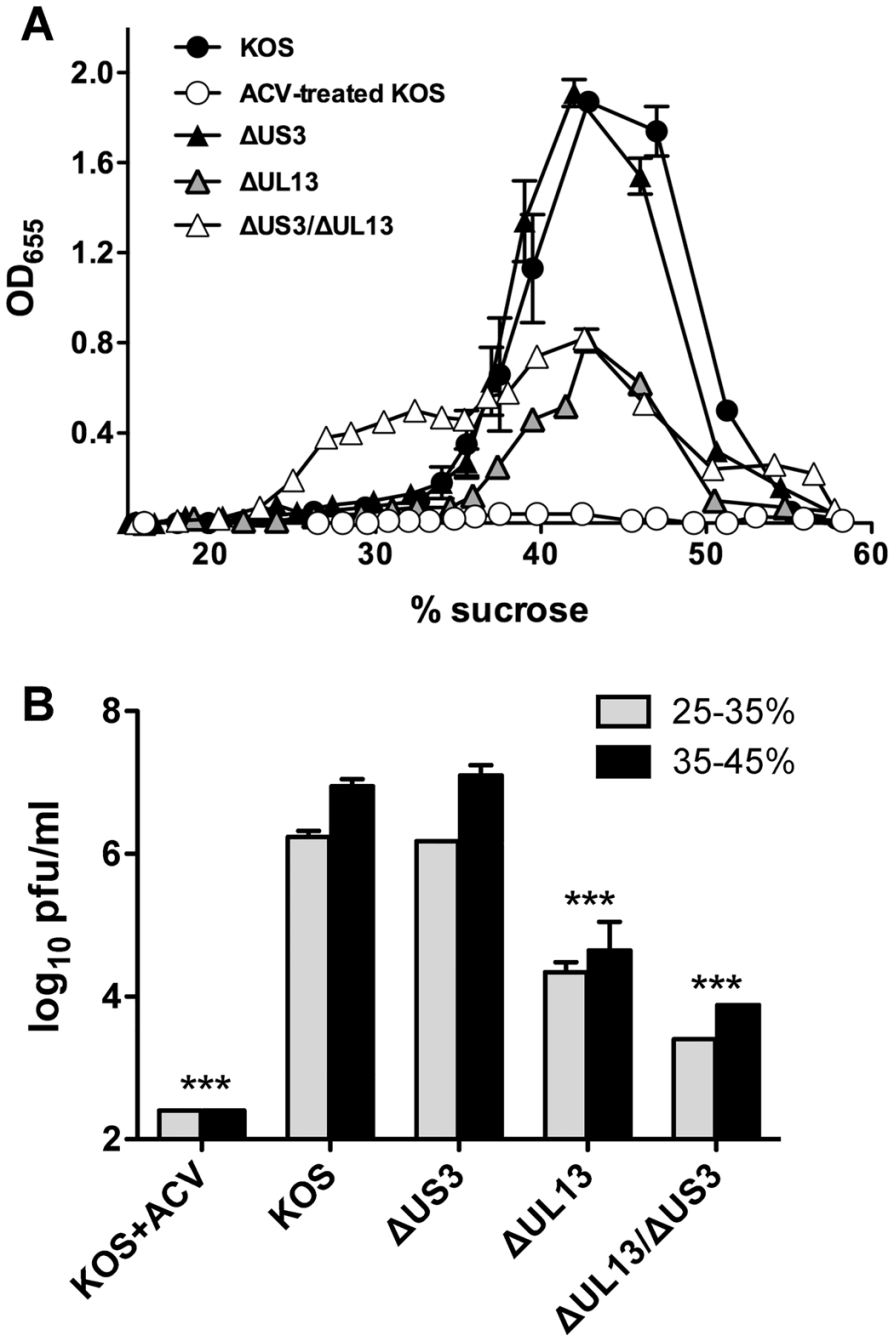
Analysis of extracellular viral particles in supernatants of Vero cells infected with HSV-1 kinase mutants. (A) Vero cells were inoculated as described in Fig 3. At 18 hours post-inoculation, the supernatants were collected and extracellular virions were purified on discontinuous sucrose gradients. Levels of total viral antigen in fractions collected from these gradients were assayed by ELISA using HRP-conjugated pan anti-HSV-1 antibody. Results are presented as intensity of OD_655_ absorbance levels relative to the uninfected Vero cells in three independent experiments. (B) 100 μl from each fraction in 25–35% and 35–45% sucrose range were pooled in viral infectivity in these pools was measured by plaque assays on Vero cells. Results presented as the mean virus yield ± SD (n = 2; ***—*p*<0.001).

The observed deficiency in the release of infectious virioins could result from a blockage of virions’ release from infected cells or from the production of defective virions. In order to distinguish between these two possibilities, the yield of total HSV-1 virions released from cells infected with HSV-1 KOS or HSV-1 kinase mutants into the culture supernatants was compared. Vero cells were inoculated with 2.5 pfu per cell for 18 hours, at which point the supernatants were removed and the HSV-1 particles purified through a discontinuous sucrose gradient. The collected fractions were used to coat 96-well EIA plates and relative abundance of HSV-1 virions evaluated by incubation with horseradish peroxidase (HRP)-conjugated anti-HSV antibody (Fig 6A). The resulting data were analyzed using two parameters: (1) area under the curve (AUC) that describes total viral antigen detected, and (2) peak value (PV) that defines the amount of viral antigen found at 40±2% sucrose, the reported buoyant density of the HSV-1 virions [94]. Acyclovir-treated, HSV-1 KOS-infected cells, which served as a negative control, produced negligible amount(s) of virus antigens measuring AUC = 2 and PV = 4 fold-increase over background. The majority of HSV-1 antigens were detected in 30–50% sucrose fractions of the HSV-1 KOS-infected supernatants (AUC = 74 and PV = 187 fold-increase over background) (Fig 6A). The yields and distribution of viral antigens in fractions from ΔUS3 supernatants were almost identical to those obtained from the HSV-1 KOS-infected cells (AUC = 68 and PV = 191 fold-increase over background) (Fig 6A). In contrast, ΔUL13 mutant virus yielded over two-fold less total virions than HSV-1 KOS (AUC = 35 and PV = 81) (Fig 6A). Likewise, the supernatant from the HSV-1 *ΔUL13/ΔUS3* mutant contained 2-fold less particles in the peak fraction (PV = 82) (Fig 6A). However, the distribution of viral antigens in supernatants obtained from this mutant differ significantly relative to the other three viruses, because significant levels of viral antigens were detected in 20–35% sucrose fractions (Fig 6A), yielding an AUC value comparable to that of the HSV-1 KOS infection (AUC = 64 vs AUC = 74, respectively). These data suggest that loss of the UL13 kinase, but not US3 kinase, correlates with the decrease in yields of total virions contributing to the overall reduction in infectivity of *ΔUL13* and *ΔUL13/ΔUS3* mutants. Importantly, a significant amount of the HSV-1 antigens detected in 20–35% sucrose fractions suggests that infection with this virus generates and releases viral components with a lower buoyant density that are not associated with mature virions (such as protein aggregates or defective/incomplete virions).

To further verify the infectivity of virions purified on sucrose gradients, their titers were measured in pooled fractions corresponding to 25–35% and 35–45% sucrose by plaque assays on Vero cells (Fig 6B). As expected, in cells infected with the wild-type HSV-1 KOS and treated with acyclovir, the titers of infectious virions were at or below the detection limits of the assay (Fig 6B). Both wild-type HSV-1 KOS and HSV-1 *ΔUS3* mutants yielded almost equivalent and high titers of infectious virions (Fig 6B). In contrast, the infectious titers of the HSV-1 *ΔUL13* mutant and even more so of the HSV-1 *ΔUL13/ΔUS3* mutant were reduced in both pooled fractions by 151-fold and 1200-fold (in 35–45% sucrose) and 77-fold and 700-fold (in 25–35% sucrose), respectively (Fig 6B). The additional 10-fold decrease in infectivity (relative to the effects shown in Fig 5) is likely a result of exposure of the virions to non-physiological conditions during purification on sucrose gradients. These data confirm that the low buoyant density HSV-1 antigens released by the HSV-1 *ΔUL13/ΔUS3* mutant and detected by ELISA (Fig 6A) are not associated with mature, infectious virions.

### Absence of herpesvirus protein kinases significantly impairs virion assembly and egress from virus-infected cells: electron microscopy analysis

**Fig 7:**
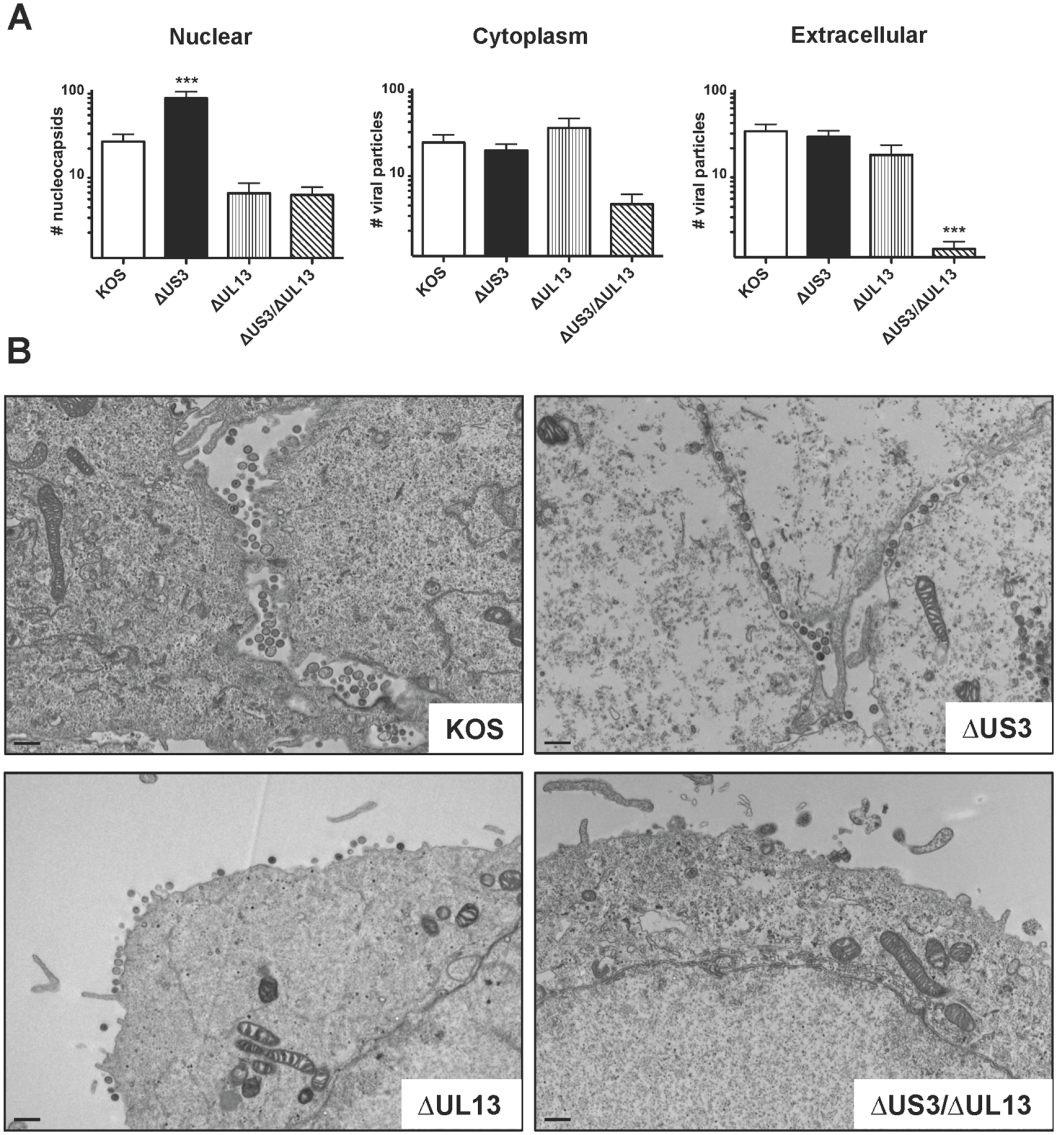
EM analysis of Vero cells infected with *ΔUS3* and *ΔUL13* HSV-1 mutants. Monolayers of Vero cells on Thermonox coverslips were inoculated as described in Fig 3. At 16 hours post-inoculation, the monolayers were fixed and processed for EM analyses. (A) Separate images of nucleus, cytoplasm, and plasma membranes/extracellular space were recorded for 20 individual cells per each virus. The number of nucleocapsids and virions were manually counted in each image in a blinded fashion. Results are presented as the mean number of virions per compartment ± sem (n = 20; ***—*p*<0.001). (B) Representative images of the plasma membranes/extracellular space for cells infected with different viruses (bars represent 500 nm).

Studies of CHPKs of human β- and γ- herpesviruses suggest that lack of their function interferes with nuclear egress as evidenced by accumulation of nucleocapsids in the nuclei of infected cells [8, 9, 11]. Likewise, the US3-deficient HSV-1 mutants exhibited a similar phenotype in electron microscopy studies [24, 33]. However, the UL13-null HSV-1 mutants have never been analyzed by the electron microscopy. To address this knowledge gap, Vero cells were inoculated with HSV-1 KOS, or HSV-1 Δ*UL13*/Δ*US3*, Δ*US3*, and Δ*UL13* mutants at MOI of 2.5 pfu per cell. At 16 hours post-inoculation the cells were fixed and processed for analysis by transmission electron microscopy (TEM). Separate images of nucleus, cytoplasm and plasma membranes/extracellular space were recorded for 20 individual cells for each virus. The number of nucleocapsids and virions were manually enumerated in each image in a blinded manner (Fig 7A). The number of nucleocapsids in the nuclei of cells infected with the *ΔUS3* mutant was significantly higher than in the nuclei of the cells infected with the HSV-1 KOS, *ΔUL13*, or *ΔUS3/ΔUL13* mutants (79±16 vs 24±5, 6±2, and 6±1 nucleocapsids, respectively) (Fig 7A, *p*<0.001). Importantly, a portion of the nucleocapsids was trapped in invaginations of the nuclear membrane, as described in previous studies [24, 33]. However, this increased accumulation of nucleocapsids had no effect on virion yields in the extracellular space and infectivity of *ΔUS3* mutant virions (Figs 5–7) corroborating a recent study by Wild et al. [95]. The HSV-1 *ΔUL13* mutant yields differed only slightly from the wild type HSV-1 KOS: the yield of nucleocapsids in the nuclei was slightly lower and the yield of virions in the cytoplasm was slightly higher. Neither of these differences was statistically significant. Most importantly, the virion yields on the membrane or extracellular space were significantly lower in cells infected with the HSV-1 *ΔUL13/ΔUS3* mutant than the cells infected with the HSV-1 KOS, or HSV-1 *ΔUS3* and *ΔUL13* mutants (1±0.3 vs 32±7, 28±5, and 17±5, respectively) (Fig 7A, *p*<0.001). These data are consistent with the micro-plaque phenotype (Fig 2) and with the apparent defect in virion release (Figs 5 and 6) during infection with the *ΔUL13* and *ΔUL13/ΔUS3* mutants. Notably, only cells infected with the HSV-1 *ΔUL13/ΔUS3* mutant often released large number(s) of membrane-bound vesicles (S1 Fig). Since one of the functions ascribed to the US3 kinase is inhibition of apoptosis [54, 66–77], it is important to emphasize that (a) release of these vesicles was not observed in cells infected with the HSV-1 *ΔUS3* mutant, (b) release of the vesicles was not accompanied by changes in chromatin structure and nuclear architecture characteristic of apoptosis, and (c) previous studies indicated that the apoptotic phenotype in HSV-1-infected cells is typically observed at later times (24 hours post-inoculation or later), whereas the experiments described here were terminated at 16 hours post-inoculation. These data suggest that the membrane-bound vesicles released during the infection with the HSV-1 *ΔUL13/ΔUS3* mutant are not consistent with the apoptotic phenotype; however, these vesicles might contain HSV-1 antigens detected in the 25–35% fractions of the sucrose gradients by ELISA (Fig 6A).

### The HSV-1 ΔUL13/ΔUS3 mutant produces significantly less infectious virus than HSV-1 ΔUL13 or ΔUS3 mutants in several cell types

**Fig 8:**
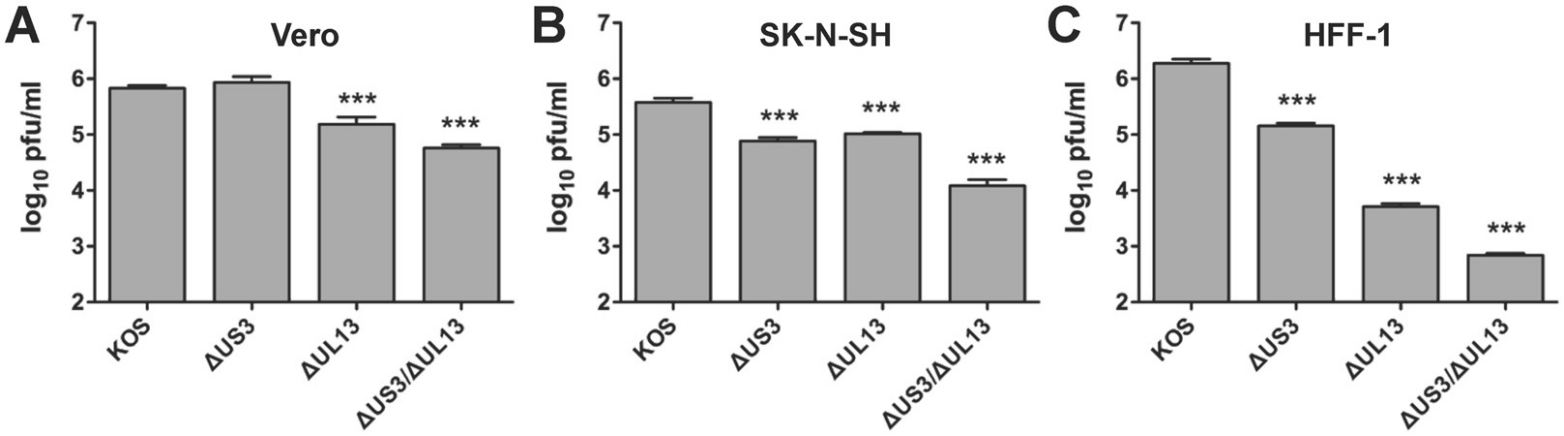
HSV-1 kinase mutants are attenuated in different cell types. Monolayers of (A) Vero, (B) SK-N-SH, and (C) HFF-1 cells were inoculated with wild type HSV-1 KOS or HSV-1 *ΔUS3*, *ΔUL13*, and *ΔUL13/ΔUS3* mutants at an MOI of 0.1 pfu per cell and incubated for 24 hours. Virus yields were determined by plaque assay on Vero cells. Results are presented as the mean virus yield ± sem (n = 6; ***—*p*<0.001).

To determine whether the effects of the loss of UL13 and US3 kinases on HSV-1 replication is cell-type-dependent, infectious virus production was verified in two additional cell types; namely, the human neuroblastoma SK-N-SH cell line (Fig 8B) and HFF-1 human foreskin fibroblasts (Fig 8C), while Vero cells served as a control (Fig 8A). Monolayers of cells were inoculated with 0.1 pfu per cell of HSV-1 KOS, or HSV-1 Δ*UL13*, Δ*US3*, and Δ*UL13*/Δ*US3* mutants and virus-infected cells were freeze-thawed at 24 hours post-inoculation to quantify viral titers in cell lysates. In Vero cells, wild-type HSV-1 KOS produced titers of 5.8 ± 0.1 log (pfu/ml) and the HSV-1 Δ*US3* mutant produced equivalent titers of 5.9 ± 0.3 log (pfu/ml) (Fig 8A). The HSV-1 Δ*UL13* mutant produced titers of 5.2 ± 0.3 log (pfu/ml), which were slightly lower than wild-type HSV-1 KOS (Fig 8A). Consistent with its micro-plaque phenotype, the HSV-1 Δ*UL13*/Δ*US3* mutant produced the lowest titers of infectious virus on Vero cells, 4.8 ± 0.1 log (pfu/ml), which were ~12-fold lower than those produced by HSV-1 KOS-infected cells (Fig 8A). In SK-N-SH cells, the HSV-1 Δ*US3* or Δ*UL13* mutant viruses produced 5- and 4-fold lower titers of infectious virus relative to wild-type HSV-1, respectively, whereas the HSV-1 Δ*UL13*/Δ*US3* mutant virus produced on average 30-fold lower titer of infectious virus (Fig 8B). In the HFF-1 cells, loss of function of either US3 or UL13 kinase significantly impaired the production of infectious virus. Specifically, in HFF-1 cells, the HSV-1 Δ*US3* mutant and Δ*UL13* mutant produced 13- and ~370-fold lower titers of infectious virus relative to wild-type HSV-1 (Fig 8C). Loss of US3 *and* UL13 kinase function had a combined effect resulting in ~2,750-fold reduction in titers of infectious virus relative to wild-type HSV-1 (Fig 8C).

Collectively, these data supported two conclusions: (a) the precise extent to which HSV-1 UL13 and US3 protein kinases contribute to infectious virus production is cell-type dependent, and (b) both HSV-1 UL13 and US3 protein kinases contribute to the production of infectious virus in each of the three cell lines tested. Notably, the loss of both kinases resulted in reduction in viral titers comparable to multiplied reductions in titers exhibited by HSV-1 mutants lacking a single protein kinase. The cell-type dependency may in part be explained by a differential expression of host cell protein kinases with overlapping substrate specificities [43, 69, 96], which may mask or attenuate the effects of viral protein kinases in certain cell types.

## Discussion

(I) The literature on CHPKs includes dozens of potential phosphorylation substrates, but does not offer any central conclusion regarding the relevance of these protein kinases to the biology of herpesviruses. Of course, we cannot exclude the possibility that the CHPK of each of the over 100 known herpesviruses phosphorylates a different set of substrates, and serves a function that is unique to the biology of each herpesvirus. However, such a hypothesis seems to contradict one of the central tenets of evolutionary biology—genes critical enough to be conserved over evolutionary time tend to fulfill the same critical role that necessitates their conservation. Therefore, it is reasonable to postulate that the conserved herpesviral kinases may play a role in the process of herpesviral replication that is (1) critical in nature, (2) conserved amongst all of the herpesviruses, and (3) has yet to be fully elucidated. Indeed, conserved protein kinases of EBV and HCMV play a critical (albeit not yet clearly defined) role in viral replication [8–11]. In contrast, the loss of UL13 kinase function yielded a modest phenotype [14, 15, 93] leading to suggestions that this protein kinase is dispensable for viral replication [14, 15, 44, 93]. However, a more detailed analysis of the HSV-1 *ΔUL13* mutant presented in this study showed that effects of the loss of UL13 function are cell type dependent and range from a modest inhibition of viral replication (7- to 15- fold relative to the wild type) to a very substantial inhibition (over 350-fold) in HFF-1 cells (Fig 8), corroborating at least one previous report [16]. This mutant also formed plaques that were 50% smaller than those formed by the wild type HSV-1 KOS (Fig 2) and released significantly less extracellular virions than the wild type virus (Fig 6). Similar to other CHPK-knockout mutants [8, 11, 13], loss of either or both HSV-1 protein kinases had no measurable effects on viral DNA accumulation (Fig 3). Moreover, despite functional differences between the HSV-1 UL13 and protein kinases encoded by human β- and γ- herpesviruses proposed in previous studies [96, 97], the loss of UL13 kinase or both kinases seems to result in a phenotype similar to that observed with mutants of other CHPKs, namely a pronounced defect in virion egress ([9, 11] and Figs 5, 6, and 8). However, the mechanisms leading to this phenotype remain unclear. While the EBV BGLF4 and HCMV UL97 protein kinases are proposed to regulate nuclear egress by localized disruption of lamina [98–100], which is consistent with their nuclear localization [101, 102], the localization of UL13 kinase has not been well defined and has been reported either as nuclear [14, 15, 103] or cytoplasmic [96, 97]. Moreover, control of the nuclear egress during HSV-1 infection seems to be mediated at least in part by the US3 kinase [21, 22, 24, 27, 33, 78–82], although an indirect effect of the UL13 kinase cannot be ruled out [36]. Alternatively, UL13 kinase could affect transcription of HSV-1 genes through its involvement in modifications of the CTD-domain of RNA Pol II [16, 44]. However, it is unlikely that such an effect would be highly selective to only a few genes (Fig 4). Thus, until the kinetics of UL13 kinase subcellular distribution is better understood and its biologically relevant partners/substrates identified, its role in viral egress will remain enigmatic.

(II) In side-by-side comparison of the HSV-1 kinase mutants in Vero cells, US3 kinase knockout had negligible effects on viral DNA accumulation, gene expression, and extracellular virion production levels (Figs 3–8), but did cause accumulation of nucleocapsids in the nucleus (Fig 7) confirming previous reports [21, 22, 33, 50, 80–82]. Notably, this mutant was moderately attenuated (13-fold) (Fig 8) in HFF cells, suggesting that the role of US3 kinase is more strictly cell-type dependent. Since this kinase is only encoded by the neurotropic α-herpesviruses, it is likely that its function can only be fully revealed in cells of neuronal origin or in primary neurons [104]. Another possible explanation for the apparent lack of significant US3 function is that a second kinase, UL13, can functionally complement its activities. Importantly, concomitant knockout of both kinases results in an enhancement of the defective phenotype (Figs 2, 5–8) in all tested cell lines.

(III) The use of the HSV-1 *ΔUL13/ΔUS3* mutant in this study demonstrated that neither US3 kinase nor UL13 kinase plays a significant role in regulation of the early stages of HSV-1 replication (Figs 3 and 4), contradicting several previous hypotheses [3, 4]. While this discrepancy may stem from cell-type dependency or differences in virus strains, the phenotype of the HSV-1 *ΔUL13* and *ΔUL13/ΔUS3* mutants parallel the phenotypes of EBV-PK- and HCMV UL97-deficient viruses [7–9, 11, 13], suggesting that the key function of all CHPKs is regulation of viral egress. Moreover, in the context of the HSV-1 *ΔUL13/ΔUS3* mutant, US3 kinase likely plays a more significant role in either virion stability or virion assembly as evidenced by additional loss of infectivity (Figs 5 and 8), reduction in levels of total extracellular virions (Figs 6 and 7) accompanied by an accumulation of viral antigens in 20–35% sucrose fractions (Fig 6) with the absence of intracellular accumulation of nucleocpsids or virions (Fig 7). However, at this juncture we cannot differentiate these two possibilities, and additional studies are warranted to address this and other questions.

### Caveats and limitations of the current study

The study examines expression levels of only a subset of viral proteins and we cannot formally claim that expression of other viral proteins was not affected by the lack of viral protein kinases’ activities. Likewise, some of the experiments were performed only at high multiplicity of infection (2.5 pfu per cell) assuming that the observed phenotype will be amplified in low MOI conditions (such as results presented in Fig 5). Since the low MOI conditions were not formally tested, we cannot rule out a possibility that the mutants may exhibit additional defects at low MOI. Finally, one of the questions that this study does not fully address is whether the functions of the HSV-1 US3 and UL13 kinases are redundant (overlapping) or independent. Our data clearly demonstrate that loss of UL13 kinase has a more profound effect on viral egress in several cell lines than does the loss of US3 kinase (Figs 2, 4–8) suggesting an unequal contribution from each of the kinases to viral egress, at least within the constrains of our experimental system. However, the data cannot distinguish between several models of synergistic genetic interactions reviewed in Perez-Perez et al. [105] and hence we cannot define the interaction between the HSV-1 kinases as fully functionally redundant (controlling one or successive steps of the same pathway), partially redundant (controlling parallel and convergent pathways), or non-redundant (controlling independent pathways). Further studies are warranted to address this question.

## Conclusion

This study presents a formalized description of the HSV-1 *ΔUL13* and *ΔUL13/ΔUS3* phenotypes. Our data establish that, even in cells where the overall effect of UL13’s loss on viral replication is moderate, virion morphogenesis and/or release are significantly affected. More importantly, these effects are greatly enhanced in the absence of both viral kinases, indicating that the protein kinases of HSV-1 act in a cooperative manner to promote viral replication. Therefore, further studies are needed to elucidate the mechanisms by which the HSV-1 UL13 and US3 protein kinases regulate viral replication and to identify biologically relevant substrates.

## Supporting Information

S1 Table PCR primers and oligonucleotide probes used in the study. (PDF)

S1 Fig Membrane-bound vesicles released by Vero cells infected with the HSV-1 ΔUL13/US3 mutant. Representative images of the plasma membranes/extracellular space for cells infected with wild type HSV-1 KOS (A) and HSV-1 ΔUL13/ΔUS3 mutant (B) (bars represent 1 μm).
(PDF)
