# Supplementary material for: The UL13 and US3 Protein Kinases of Herpes Simplex Virus 1 Cooperate to Promote the Assembly and Release of Mature, Infectious Virions
Source: PLoS One. 2015 Jun 26;10(6):e0131420. doi: 10.1371/journal.pone.0131420 (PMC4482649; doi:10.1371/journal.pone.0131420)
Supplement: S1 Table — (PDF) [file pone.0131420.s001.pdf]

Primer/probe	Name	Application
CAGGTACCTACGTACACTCCAACC	UL13_1	UL13 mutagenesis
CTTGGATCCATATGGGATCTCGAGGGTCGTGTAG	UL13_2	UL13 mutagenesis
CTTGGATCCACGCGTGTCACCCTCAACTCCAAC	UL13_3	UL13 mutagenesis
CTATCTAGAGAAGTCCGGCGACAGCTG	UL13_4	UL13 mutagenesis
GCGACCTGGCCGAAGGATGGATGAG	a	PCR test (Fig. 1)
CTTGTACAGCTCGTCCATG	b	PCR test (Fig. 1)
GGGAGGTCTATATAAGCAGAGCT	c	PCR test (Fig. 1)
CCGGGTGAACGCTGATTCTCACGAC	d	PCR test (Fig. 1)
GTCCGTTGTGTTGGCC	e	PCR test (Fig. 1)
CGGTCTCTCTCGAGATACATTGATGAGTTTG	f	PCR test (Fig. 1)
AATGGTGAGCAAGGGCG	g	PCR test (Fig. 1)
CAGCGACAGGACCTAG	h	PCR test (Fig. 1)
AGGCCCCCAGAGACTTGTTGTAGGAGCATTCGGTGTACTC	US6 probe	Dot-blot
CCGTGGGGGTTGGAGTGTACGTAGGATGCGAGCCAATCCT	UL13 probe	Southern blot
TCCTCTATGGGGTAGTCCTGGTTTCCGTACATCTGGGCA	US3 probe	Southern blot
