# Supplementary figures and images for: The UL13 and US3 Protein Kinases of Herpes Simplex Virus 1 Cooperate to Promote the Assembly and Release of Mature, Infectious Virions
Source: PLoS One. 2015 Jun 26;10(6):e0131420. doi: 10.1371/journal.pone.0131420 (PMC4482649; doi:10.1371/journal.pone.0131420)

**A**

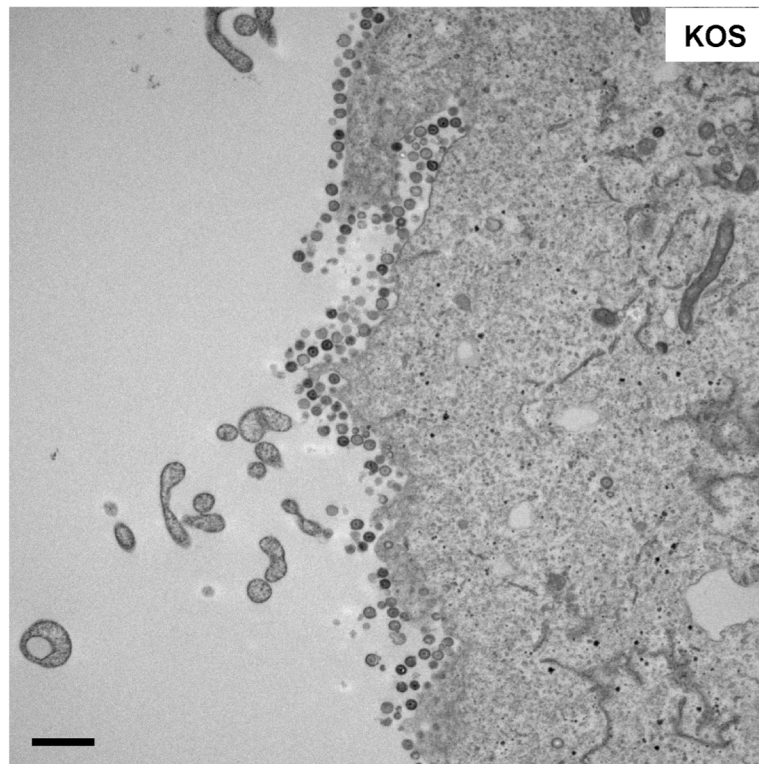

**B**

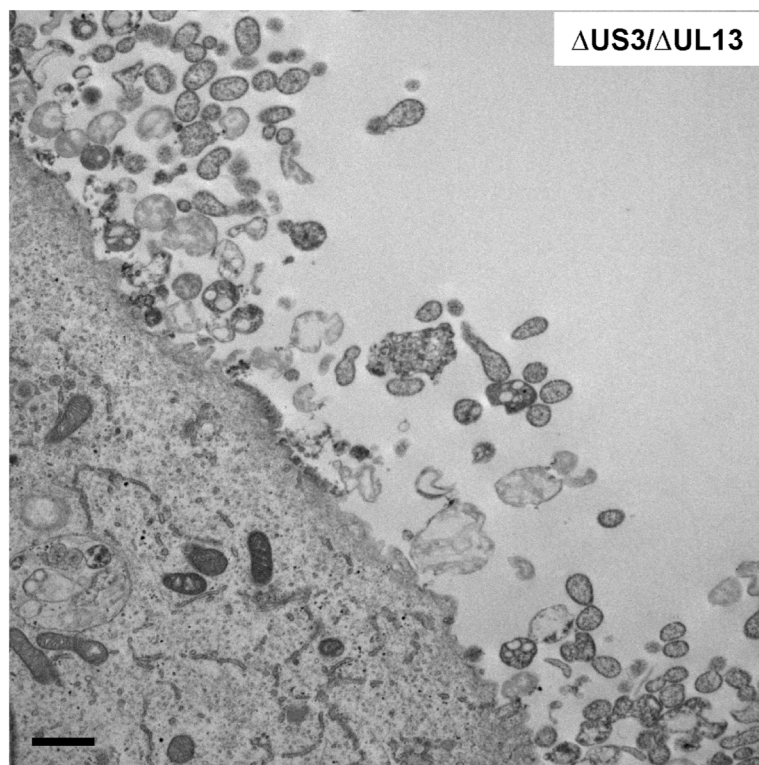

Supplement: S1 Fig — Representative images of the plasma membranes/extracellular space for cells infected with wild type HSV-1 KOS (A) and HSV-1 ΔUL13/ΔUS3 mutant (B) (bars represent 1 μm). (PDF) [file pone.0131420.s002.pdf]
